# Skin diseases of the nipple and areola complex: A case series study from China

**DOI:** 10.3389/fmed.2023.1136482

**Published:** 2023-03-28

**Authors:** Chao Wu, Qian-Nan Jia, Kai Fang, Yue-Ping Zeng

**Affiliations:** Department of Dermatology, State Key Laboratory of Complex Severe and Rare Diseases, Peking Union Medical College Hospital, Chinese Academy of Medical Sciences and Peking Union Medical College, National Clinical Research Center for Dermatologic and Immunologic Diseases, Beijing, China

**Keywords:** skin diseases, nipple and areola complex, adenoma of the nipple, breast, eczema

## Abstract

**Background:**

Skin diseases of the nipple and areola complex (NAC) are numerous and difficult to diagnose, which is a great challenge for clinicians. A better understanding of the clinical features of NAC skin diseases is of great value for the correct diagnosis.

**Methods:**

To investigate the clinical characteristics of skin diseases of the NAC, we retrospectively analyzed the demographic data, disease constitution, rash characteristics, inconsistency between the clinical and pathological diagnosis from 260 patients with NAC lesions that were confirmed by histopathology at Peking Union Medical College Hospital, China from 2012 to 2022.

**Results:**

The patients’ average age was 43.6 (8 to 82) years, and the ratio of females to males was 13.4:1. Out of the 260 patients biopsied, the most common diseases were eczema, Paget’s disease (PD), adenoma of the nipple (AN), seborrheic keratosis (SK), cutaneous metastasis of breast cancer, wart, soft fibroma, and hyperkeratosis of the nipple and areola. There were 77 (29.6%) patients with inconsistency between the clinical impressions and pathological diagnoses. AN was the most clinically misdiagnosed condition, most commonly presumed to be PD or eczema.

**Conclusion:**

Eczema and PD are the most common biopsied NAC skin diseases. Late onset, unilateral involvement, and predilection for the nipple are several characteristics of PD, which are different from eczema. NAC skin diseases are easily misdiagnosed clinically, especially AN.

## 1. Introduction

Skin diseases of the nipple and areola complex (NAC) are numerous and difficult to diagnose, which is a great challenge for clinicians (1, 2). A variety of NAC skin tumors often share similar clinical manifestations with inflammatory skin diseases, so they are easily missed or misdiagnosed (3, 4). In addition, because of the unusual location of the diseases, patients are often reluctant to see the doctor. Delayed treatment often greatly

affects the prognosis. Although histopathological examination can confirm the diagnosis, a skin biopsy may influence the appearance and function of the NAC (5). Therefore, a better understanding of the clinical features of NAC skin diseases is of great value for the correct diagnosis. To improve dermatologists’ understanding of the clinical features of NAC lesions, we retrospectively analyzed the demographic data, disease constitution, rash characteristics, and inconsistency between the clinical and pathological diagnosis from 260 patients. These patients had NAC skin diseases that were confirmed by skin histopathology at Peking Union Medical College Hospital from October 2012 to October 2022.

## 2. Materials and methods

In this retrospective study, we included 260 patients with NAC lesions that were confirmed by skin histopathology at Peking Union Medical College Hospital, China, from October 2012 to October 2022. The demographic data, disease constitution, rash characteristics, and inconsistency between the clinical and pathological diagnosis were analyzed retrospectively. This study was approved by the ethics committee of Peking Union Medical College Hospital.

## 3. Results

### 3.1. General information

The patients’ mean age was 43.6 years, and ranged from 8 to 82 years. There were 242 females, aged 10–82 years, with an average age of 44.0 years. There were 18 males, aged 8–82 years, with an average age of 37.7 years. The ratio of females to males was 13.4:1. The number of patients in each age group is presented in Table 1.

**TABLE 1:** Clinical features of the most common biopsied skin diseases of the nipple and areola complex.

	Eczema	Paget’s disease	Adenoma of the nipple	Seborrheic keratosis	Breast cancer cutaneous metastasis	Wart	Soft fibroma	Hyperkeratosis of the nipple and areola
Total cases	94	80	16	11	9	9	7	6
Average age (year)	36.67	52.68	40.13	45.82	61.89	45.22	38.29	37
Age range (year)	8–82	25–81	21–51	27–73	42–77	22–82	25–50	25–68
Unilateral (*n*)	67	80	14	11	9	9	7	1
Bilateral (*n*)	27	0	2	0	0	0	0	5
Nipple (*n*)	34	65	15	0	6	8	6	2
Areola (*n*)	40	7	0	10	1	1	1	2
Nipple and areola (*n*)	20	8	1	1	2	0	0	2
Patch/macules (*n*)	82	70	12	4	6	0	0	2
Papule/nodule/plaque (*n*)	12	10	4	7	3	9	7	4
Exudation/erosion (*n*)	40	54	9	1	6	1	0	0
Course range (month)	0.23–240	1–120	0.7–72	6–360	0.23–24	2–120	0.33–108	1–120
Average course (month)	15.34	17.04	18.48	58.55	7.69	39.67	34.48	44.17
Clinical misdiagnosis (%)	14 (14.89)	21 (26.2)	14 (87.5)	4 (36.36)	3 (33.33)	3 (33.33)	3 (42.86)	1 (16.6)

### 3.2. Disease constitution, age distribution, and gender distribution

**FIGURE 1:**
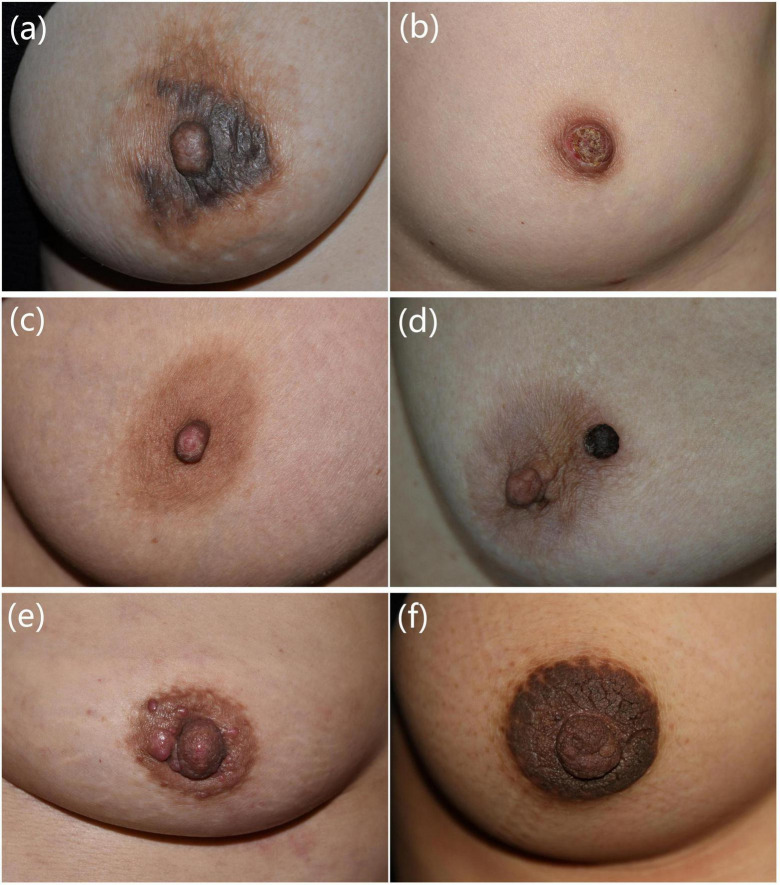
Clinical manifestations of common nipple and areola complex skin diseases. **(a)** A 40-year-old female presented with erythema, papules, exudation with itching of bilateral areolas for 1 month. Eczema was diagnosed. **(b)** A 49-year-old female presented with erythema, erosion, and discharge on the right nipple for 1 year. Paget’s disease was diagnosed. **(c)** A 45-year-old female presented with erosion, scales, and bloody exudation of the left nipple for 2 years. Adenoma of the nipple was diagnosed. **(d)** A 63-year-old female presented with a black nodule on the left areola for 2 months. Seborrheic keratosis was diagnosed. **(e)** A 53-year-old female presented with red nodules on the left nipple and areola for 3 months. Cutaneous metastasis of breast cancer was diagnosed. **(f)** A 26-year-old female presented with hyperpigmented and verrucous plaques of bilateral nipples and areolas for 10 years. Hyperkeratosis of the nipple and areola was diagnosed. Pathological manifestations of these patients are presented in Supplementary Figure 1.

Out of the 260 patients biopsied, 36.2% had eczema, 30.8% had Paget’s disease (PD), 6.2% had adenoma of the nipple (AN), 4.2% had seborrheic keratosis (SK), 3.5% had cutaneous metastasis of breast cancer, 3.5% had warts, 2.7% had soft fibroma, and 2.3% had hyperkeratosis of the nipple and areola. Clinical manifestations of these diseases are presented in Figure 1 and Table 1. Pathological manifestations of these diseases are presented in Supplementary Figure 1. Other miscellaneous diseases included are presented in Table 2. Eczema was the most common disease in the 0–18 years, and 19–50 years age groups. In the age group older than 50 years, PD was the most common disease. The most common biopsied NAC skin diseases in female patients were eczema (35.5%), PD (32.6%), and AN (6.6%). The most common biopsied NAC skin diseases in male patients were eczema (44.4%), wart (27.8%), and SK (11.1%). The age and gender distribution is presented in Table 2.

**TABLE 2:** Disease constitution, age distribution, and gender distribution.

Disease types	Total	∼18 years 21 cases		19∼50 years 143 cases		>50 years 96 cases	
		F	M	F	M	F	M
**Benign tumors**							
Adenoma of the nipple	16			15		1	
Seborrheic keratosis	11			5	1	4	1
Wart	9			4	1		4
Soft fibroma	7			6		1	
Hyperkeratosis of the nipple and areola	6			5		1	
Sebaceous hyperplasia	2			2			
Keloid	1			1			
Neurofibroma	1			1			
Fibroma	1			1			
Eccrine hidrocystoma	1			1			
Melanoacanthoma	1					1	
Dilated pore	1					1	
Leiomyoma	1				1		
Melanocytic nevus	3	1	1	1			
Lentigo	2	1		1			
**Malignant tumors**							
Paget’s disease	80			30	1	49	
Cutaneous metastasis of breast cancer	9			1		8	
Squamous cell carcinoma	1			1			
Primary cutaneous CD30 positive large cell anaplastic lymphoma	1			1			
Malignant melanoma	1				1		
Bowen disease	1					1	
**Inflammatory and infectious diseases**							
Eczema	94	14	3	51	5	21	
Syphilis	2	1		1			
Non-specific infections	2			1		1	
Lichen planus	2			1		1	
Contact dermatitis	1			1			
Lichen sclerosus	1			1			
Cutaneous amyloidosis	1			1			
Pemphigus vulgaris	1					1	

### 3.3. Rash characteristics

#### 3.3.1. Symmetry and distribution

Nineteen types of skin diseases occurred only in unilateral breasts. Six types of skin disease occurred only in bilateral breasts. There were four types of skin disease that can occur either in unilateral breast or bilateral breasts (Table 3). Five types of skin diseases occurred only in the nipple. Eleven types of skin diseases occurred only in the areola. Thirteen types of skin diseases could occur either in the nipple or the areola (Table 4).

**TABLE 3:** Symmetry of skin diseases of the nipple and areola complex.

Disease types	Total	Unilateral	Bilateral
**Benign tumors**			
Adenoma of the nipple	16	14	2
Seborrheic keratosis	11	11	
Wart	9	9	
Soft fibroma	7	7	
Hyperkeratosis of the nipple and areola	6	1	5
Sebaceous hyperplasia	2		2
Keloid	1	1	
Neurofibroma	1		1
Fibroma	1	1	
Eccrine hidrocystoma	1	1	
Melanoacanthoma	1	1	
Dilated pore	1	1	
Leiomyoma	1		1
Melanocytic nevus	3	3	
Lentigo	2	2	
**Malignant tumors**			
Paget’s disease	80	80	
Cutaneous metastasis of breast cancer	9	9	
Squamous cell carcinoma	1	1	
Primary cutaneous CD30 positive large cell anaplastic lymphoma	1	1	
Malignant melanoma	1	1	
Bowen disease	1	1	
**Inflammatory and infectious diseases**			
Eczema	94	67	27
Syphilis	2	1	1
Non-specific infections	2	2	
Lichen planus	2	2	
Contact dermatitis	1		1
Lichen sclerosus	1	1	
Cutaneous amyloidosis	1		1
Pemphigus vulgaris	1		1

**TABLE 4:** Distribution of skin diseases of the nipple and areola complex.

Disease types	Total	Nipples	Areolas	Nipples and areolas
**Benign tumors**				
Adenoma of the nipple	16	15		1
Seborrheic keratosis	11		10	1
Wart	9	8	1	
Soft fibroma	7	6	1	
Hyperkeratosis of the nipple and areola	6	2	2	2
Sebaceous hyperplasia	2		2	
Keloid	1		1	
Neurofibroma	1	1		
Fibroma	1		1	
Eccrine hidrocystoma	1		1	
Melanoacanthoma	1		1	
Dilated pore	1	1		
Leiomyoma	1		1	
Melanocytic nevus	3	2	1	
Lentigo	2	1	1	
**Malignant tumors**				
Paget’s disease	80	65	7	8
Cutaneous metastasis of breast cancer	9	6	1	2
Squamous cell carcinoma	1	1		
Primary cutaneous CD30 positive large cell anaplastic lymphoma	1		1	
Malignant melanoma	1		1	
Bowen disease	1		1	
**Inflammatory and infectious diseases**				
Eczema	94	34	40	20
Syphilis	2	2		
Non-specific infections	2	2		
Lichen planus	2	1	1	
Contact dermatitis	1			1
Lichen sclerosus	1		1	
Cutaneous amyloidosis	1		1	
Pemphigus vulgaris	1			1

#### 3.3.2. Rash characteristics of common biopsied diseases

##### 3.3.2.1. Eczema

About two-thirds of the rashes occurred in unilateral breasts, and they could occur in the nipple alone, areola alone, or nipple and areola at the same time. The rashes were mainly patches or macules, and 42.6% of the patients had exudation or erosion (Table 1).

##### 3.3.2.2. Paget’s disease

All the PD rashes occurred in unilateral breasts, and most of them only occurred in the nipple. The rashes were mainly patches or macules, and 67.5% of the patients had exudation or erosion (Table 1).

##### 3.3.2.3. Adenoma of the nipple

Fourteen patients had unilateral AN and two patients had bilateral AN. The lesions in 15 patients were located in the nipple, and in one patient, it was located in the nipple and areola. The average disease course before visit our department was 18.48 months (Table 1).

#### 3.3.3. Inconsistency between the clinical and pathological diagnosis

Among the 260 patients, there were 77 (29.6%) patients with inconsistency between the clinical impressions and pathological diagnoses. AN, soft fibroma, SK, cutaneous metastasis of breast cancer, wart, PD, hyperkeratosis of the nipple and areola, and eczema were diseases with high inconsistent rate between the clinical impressions and pathological diagnoses. A total of 87.5% of patients diagnosed histologically as AN were clinically considered as other diseases, which was the most easily misdiagnosed clinically.

## 4. Discussion

To the best of our knowledge, our study is the largest single-center study of NAC skin diseases to date. We retrospectively analyzed the demographic data, disease constitution, rash characteristics, and inconsistency between the clinical and pathological diagnosis from 260 patients with NAC lesions that were confirmed by histopathology at Peking Union Medical College Hospital. Cinotti et al. retrospectively analyzed clinical data from 131 patients with NAC lesions from 13 hospitals in Italy (6). Their study included the following patient distribution: three with MM, 15 with PD, 66 with melanocytic nevus, seven with melanosis, 16 with SK, ten with eczema, and 14 with miscellaneous lesion (three with AN, one with angioma, one with bullous pemphigoid, one with epidermal cyst, one with Fordyce spots, one with hematoma, two with melanoacanthoma, one with mycosis fungoides, one with pigmented Bowen disease, one with radiodermatitis, and one with xerosis). The reasons of different disease spectrum between this two studies may be due to different research purposes and inclusion criteria. Our study was mainly to analyze the disease constitution and clinical characteristics of NAC lesions, and all diagnoses were confirmed by skin histopathology. However, Cinotti et al. focused on analyzing dermoscopic and confocal microscopic features of NAC lesions, and the enrolled patients were confirmed by skin histopathology or greater than a 1-year follow-up.

In our study, the most common biopsied NAC skin diseases were eczema and PD. PD is a relatively rare breast disease with an incidence of 1–3% among all breast cancers, which is often manifested as intraductal carcinoma *in situ* or invasive ductal carcinoma (7, 8). Clinical manifestations include itching, erythema, scales, erosion, ulcer, bloody secretion, or nipple retraction (9, 10). In the early stages, PD is often misdiagnosed as eczema, resulting in delayed treatment. In our study, late onset, unilateral involvement and predilection for the nipple are several characteristics of PD, which are different from eczema.

It has been reported that an age of 40–50 years is the peak time for benign breast diseases, and the incidence of malignant breast diseases increases after menopause (11). In the present study, the disease constitution in young patients aged 0–18 years were eczema, melanocytic nevus, lentigo, and syphilis, all of whom had benign diseases. This suggests that dermoscopy and other non-invasive examinations should be the first choice for young patients, and invasive examinations such as skin biopsy should be minimized to avoid affecting the development of nipple and areola in adolescents (12). For malignant diseases, there were 80 patients with PD in the present study. Among them, 29 were patients aged 30–49 years, and premenopausal patients accounted for about 36% of these patients. There were nine patients with cutaneous metastasis of breast cancer, among whom eight patients were over 50 years old. This indicated that cutaneous metastasis of breast cancer was more common after menopause. Other malignancies in the present study included Bowen disease, MM, primary cutaneous CD30-positive large cell anaplastic lymphoma, and SCC, which occurred in patients who were 56, 46, 37, and 35 years old, respectively. The onset age of NAC malignancies in our study was earlier than that reported in the literature, suggesting that clinicians should be alert to the possibility of malignant NAC lesions in patients over 30 years of age.

There are many kinds of skin tumors that occur in the nipple and areola, including benign tumors and malignant tumors (13). Spyropoulou et al. reviewed 337 previous publications on benign tumors of the nipple, including neurofibroma, leiomyoma, milium, AN, syringomatous adenoma, nevoid hyperkeratosis, fibroma, pseudolymphoma, and hemangioma (11). Benign tumors in the present study also included SK, soft fibroma, melanoacanthoma, eccrine hidrocystoma, dilated pore, keloid, sebaceous gland hyperplasia, lentigo, and melanocytic nevus. Reported malignant tumors included breast cancer, PD, mycosis fungoides, cancer cutaneous metastasis, lymphoma, sarcoma, SCC, basal cell carcinoma, and MM (14, 15). The malignancies in our study included PD, breast cancer cutaneous metastasis, Bowen disease, SCC, MM, and primary cutaneous CD30-positive large-cell anaplastic lymphoma. Both benign and malignant tumors can manifest as pruritus, exudation, lichenification, erosion, and nodular hyperplasia. It is difficult to accurately diagnose using imaging, and histopathology is the golden standard.

In addition to neoplastic skin diseases, there are relatively few reports on other kinds of NAC skin diseases. Inflammatory and infectious NAC skin diseases include atopic dermatitis, contact dermatitis, radiation dermatitis, psoriasis, syphilis, wart, atypical mycobacteria infection, hidradenitis suppurativa, intertrigo, confluent and reticulated papillomatosis, Fox–Fordyce disease, and pyoderma gangrenosum (16). Special diseases found in the present study included lichen planus, lichen sclerosis, primary cutaneous amyloidosis, and pemphigus vulgaris, which were rarely reported in the literature.

There were 18 male patients in the present study, and their disease spectrum was significantly different from that of female patients. The NAC skin diseases in males are mainly benign. Benign masses in the male breast include gynecomastia, epidermoid cyst, lipoma, intraductal papilloma, pseudohemangiomatous stromal hyperplasia, granulosa cell tumor, hemangioma, schwannoma, myofibroblastoma, and fibromatosis (17). Malignant skin diseases in the male breast are rare. Breast cancer cutaneous metastasis (17), PD (18), and basal cell carcinoma (19) have been reported. Unlike other malignancies, basal cell carcinoma of the breast is more common in men compared with women, which may be related to the exposure to sunlight for the male chest (19).

Nipple and areola complex skin diseases are easily misdiagnosed clinically. In our study, 87.5% of patients diagnosed histologically as AN were clinically considered as other diseases. AN is a rare benign epithelial neoplasm that occurs in the papillary ducts, and it mainly affects women aged 43 to 45 years, although it has also been reported in men and adolescents (20). Clinically, AN often manifests as unilateral nipple erythema, swelling, erosion, and exudation, which needs to be distinguished from cutaneous metastasis of breast cancer, PD, and eczema (21). If the patient is misdiagnosed with a malignant tumor, the extent of surgical resection is much larger, and the harm is great (22).

Dermoscopy and reflectance confocal microscopy (RCM) are widely used techniques for the diagnosis of skin diseases, and their diagnostic performance for NAC lesions has recently been made (1). The dermoscopic and reflectance confocal microscopic characteristics of the most common four diseases in our study are presented in Table 5 and Supplementary Figure 2. The dermoscopic features of eczema are linear vessels, scattered dotted vessels, and yellow scales (8). Histopathologically, linear vessels correspond to dilated vessels in the dermis parallel to the skin surface, mostly located below the dermal papillary layer. Dotted vessels correspond to apical dilated vessels of the dermal papilla. Yellow scales correspond to parakeratosis and hyperkeratosis. The most frequent dermoscopic criteria of non-pigmented MPD are pink structureless areas, white lines, dotted vessels, erosion/ulceration and white scales. The most frequent dermoscopic criteria of pigmented MPD are gray granules/dots, pink structureless areas and white lines (8). Vessels at dermoscopy correlate with dilated vessels at histopathology, but their shape can differ because the epidermis can have a squamous or a papillomatous hyperplasia with a consequent different orientation of the underlying vessels (1). Multiple blue-gray dots (peppering) correspond to melanophages in the papillary dermis, while the white scar areas to fibrosis (23). The shiny white streaks, originally termed chrysalis-like structures. The lines are generally oriented parallel or orthogonally to each other. These structures represent new or remodeled collagen bundles (23). The dermoscopic features of AN are whitish/yellowish hyperkeratosis, cherry-red dotted and linear vessels on a pinkish background or increased red serpiginous and annular structures. Histopathologically, these dermoscopic characteristics correspond to luminal openings and remaining epidermis (24). Most cases of SK exhibit the typical dermoscopic findings of fissures and ridges, hairpin vessels with white halo, comedo-like openings, and milia-like cysts. Histopathologically, these dermoscopic characteristics correspond to papillomatous surface of the epidermis, enlarged capillaries of the dermal papillae, pseudohorn cysts in the epidermis opened to the surface of the lesion and intraepidermal cysts, respectively (25).

**TABLE 5:** Dermoscopic and reflectance confocal microscopic characteristics of the most common four diseases in the study.

Diagnosis	Characteristics of dermoscopy	Characteristics of reflectance confocal microscopy
Eczema	Linear vessels, scattered dotted vessels, yellow scales (8)	Vesicles with necrotic keratinocytes in the epidermis, spongiosis (1)
Paget’s disease	Non-pigmented MPD: pink structureless areas, white lines, dotted vessels, erosion/ulceration, white scales Pigmented MPD: gray granules/dots, pink structureless areas, white lines (8)	Disorganized epidermis, multiple hyper-reflective cells of varying size and shape, large hyporeflective cells in the epidermis in single units or in nests, inflammatory cells in the epidermis and dermis (1)
Adenoma of the nipple	Pinkish background, whitish/yellowish hyperkeratosis, cherry-red dotted and linear vessels, red serpiginous and annular structures (1, 24)	None reported in the literature.
Seborrheic keratosis	Fissures and ridges, hairpin vessels with white halo, comedo-like openings, milia-like cysts (25)	Epidermal brain-like structure, bright dermal papillary rings, looped vessels at the abnormal dermal papilla (26)

In conclusion, we presented a large single-center study of biopsied NAC skin diseases. Eczema and PD are the most common biopsied skin diseases of the NAC. Late onset, unilateral involvement, and predilection for the nipple are several characteristics of PD, which are different from eczema. NAC skin diseases are easily misdiagnosed clinically, especially AN. A better understanding of the clinical features of NAC skin diseases is of great value for the correct diagnosis.

## Data availability statement

The data analyzed in this study is subject to the following licenses/restrictions: The dataset is in the data system of Peking Union Medical College Hospital and cannot be exported or disclosed. Requests to access these datasets should be directed to Y-PZ, zengyueping0917@126.com.

## Ethics statement

The studies involving human participants were reviewed and approved by Ethics Committee of Peking Union Medical College Hospital. The patients/participants provided their written informed consent to participate in this study.

## Author contributions

CW wrote the manuscript. Q-NJ and KF collected patients’ data. Y-PZ revised the manuscript. All authors contributed to the article and approved the submitted version.

## Supplementary material

SUPPLEMENTARY FIGURE 1 Pathological manifestations of common nipple and areola complex skin diseases. **(A)** Histopathology of the patient in Figure 1A revealed hyperkeratosis, parakeratosis, irregular acanthosis, intercellular edema in stratum spinosum, and a small number of lymphocytes infiltrating around the vessels in the superficial dermis (hematoxylin-eosin, bar = 500 μm). **(B)** Histopathology of the patient in Figure 1B revealed scattered or nest-like distributed large cells in the epidermis. The large cells exhibited pleomorphic vesicular nuclei with prominent nucleoli and pale cytoplasm (hematoxylin-eosin, bar = 500 μm). **(C)** Histopathology of the patient in Figure 1C revealed many tumor cell masses without capsule in the dermis. The tumor cells were arranged into tubular structures, with an internal layer of cuboidal epithelial cells with an apocrine secretion and an external layer of myoepithelial cells (hematoxylin-eosin, bar = 2 mm). **(D)** Histopathology of the patient in Figure 1D revealed orthokeratosis, acanthosis, horn cysts and horn pseudocyst in the epidermis (hematoxylin-eosin, bar = 4 mm). **(E)** Histopathology of the patient in Figure 1E revealed a large number of tumor cell masses in the dermis, mainly located in the tubular structure. Diffuse heteromorphic tumor cells were arranged into glandular structures (hematoxylin-eosin, bar = 1 mm). **(F)** Histopathology of the patient in Figure 1F revealed orthokeratosis, acanthosis, and pigmentation of the basal layer (hematoxylin-eosin, bar = 1 mm).

SUPPLEMENTARY FIGURE 2 Dermoscopic manifestations of common nipple and areola complex skin diseases. **(A)** Dermoscopic examination of eczema of the nipple revealed linear vessels, scattered dotted vessels, and yellow scales. **(B)** Dermoscopic examination of mammary Paget’s disease revealed pink-whitish areas, pigmented network, linear and dotted vessels, streaks, and scales. **(C)** Dermoscopic examination of adenoma of the nipple revealed whitish/yellowish hyperkeratosis, bright white stripes, and dotted vessels on a pinkish background. **(D)** Dermoscopic examination of seborrheic keratosis of the areola revealed clear boundary, comedo-like openings, and hairpin vessels.
