# Supplementary figures and images for: Skin diseases of the nipple and areola complex: A case series study from China
Source: Front Med (Lausanne). 2023 Mar 28;10:1136482. doi: 10.3389/fmed.2023.1136482 (PMC10086167; doi:10.3389/fmed.2023.1136482)

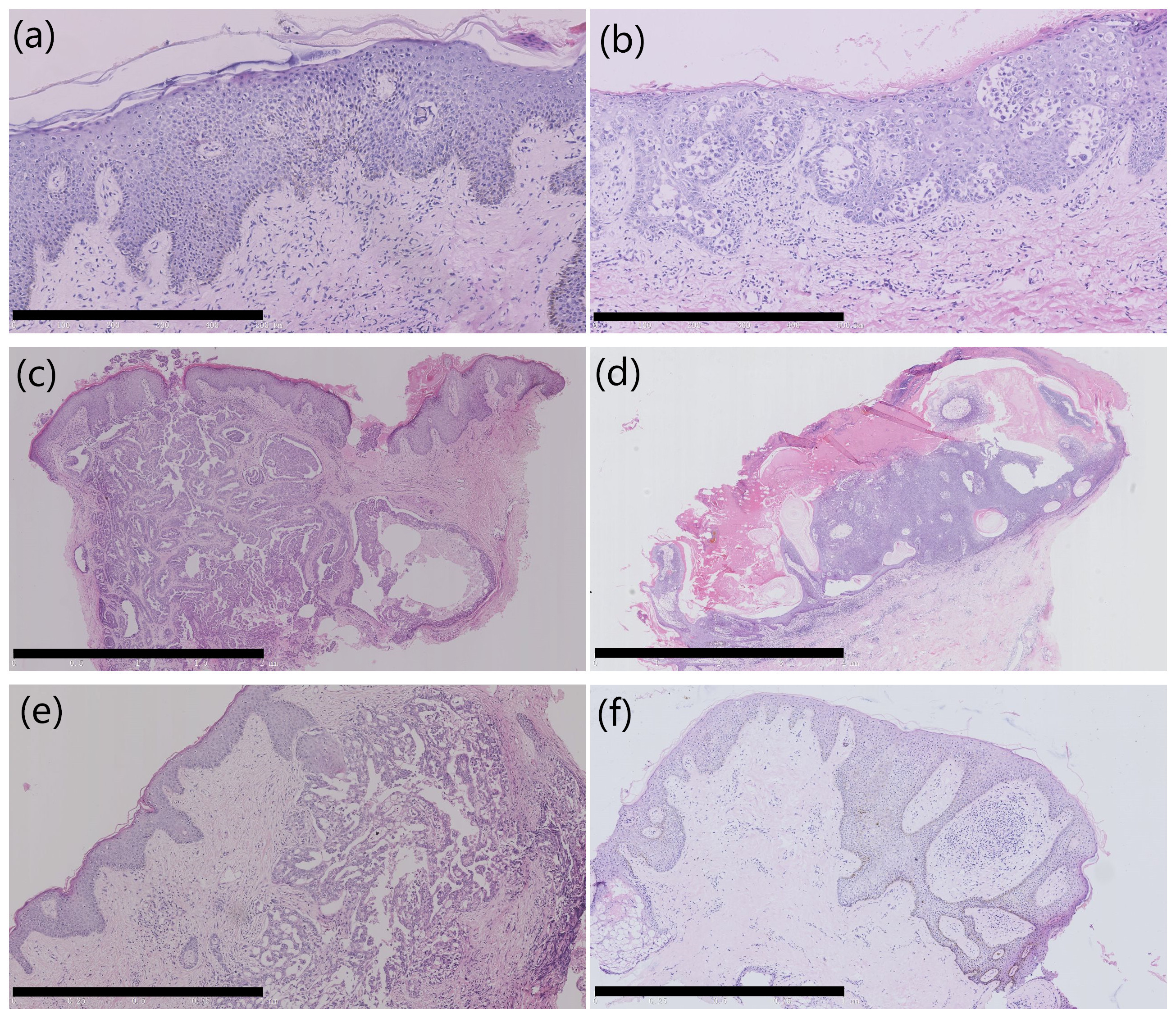

Supplement: SUPPLEMENTARY FIGURE 1 — Pathological manifestations of common nipple and areola complex skin diseases. (A) Histopathology of the patient in Figure 1A revealed hyperkeratosis, parakeratosis, irregular acanthosis, intercellular edema in stratum spinosum, and a small number of lymphocytes infiltrating around the vessels in the superficial dermis (hematoxylin-eosin, bar = 500 μm). (B) Histopathology of the patient in Figure 1B revealed scattered or nest-like distributed large cells in the epidermis. The large cells exhibited pleomorphic vesicular nuclei with prominent nucleoli and pale cytoplasm (hematoxylin-eosin, bar = 500 μm). (C) Histopathology of the patient in Figure 1C revealed many tumor cell masses without capsule in the dermis. The tumor cells were arranged into tubular structures, with an internal layer of cuboidal epithelial cells with an apocrine secretion and an external layer of myoepithelial cells (hematoxylin-eosin, bar = 2 mm). (D) Histopathology of the patient in Figure 1D revealed orthokeratosis, acanthosis, horn cysts and horn pseudocyst in the epidermis (hematoxylin-eosin, bar = 4 mm). (E) Histopathology of the patient in Figure 1E revealed a large number of tumor cell masses in the dermis, mainly located in the tubular structure. Diffuse heteromorphic tumor cells were arranged into glandular structures (hematoxylin-eosin, bar = 1 mm). (F) Histopathology of the patient in Figure 1F revealed orthokeratosis, acanthosis, and pigmentation of the basal layer (hematoxylin-eosin, bar = 1 mm). [file Figure_1.TIF]

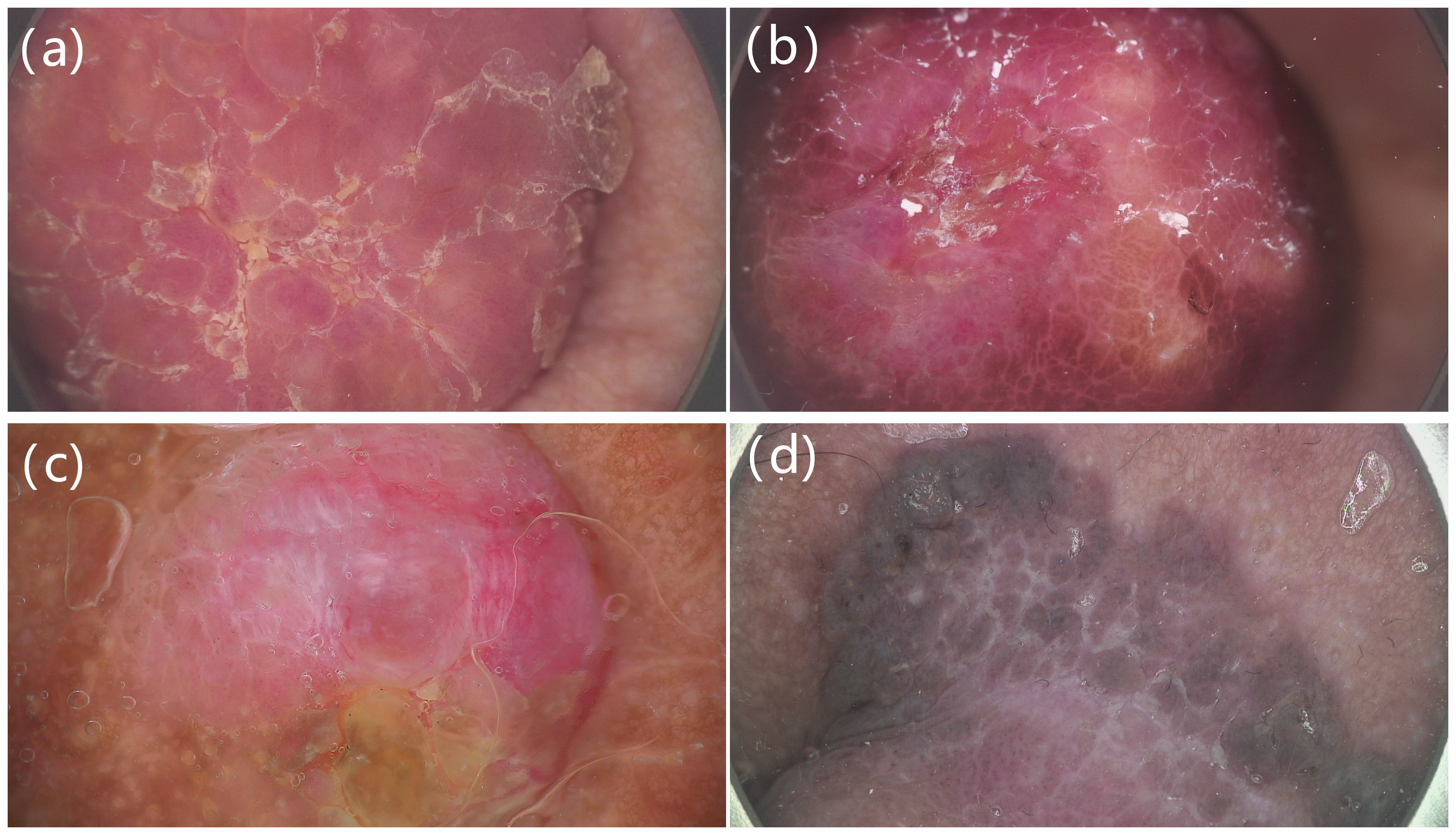

Supplement: SUPPLEMENTARY FIGURE 2 — Dermoscopic manifestations of common nipple and areola complex skin diseases. (A) Dermoscopic examination of eczema of the nipple revealed linear vessels, scattered dotted vessels, and yellow scales. (B) Dermoscopic examination of mammary Paget’s disease revealed pink-whitish areas, pigmented network, linear and dotted vessels, streaks, and scales. (C) Dermoscopic examination of adenoma of the nipple revealed whitish/yellowish hyperkeratosis, bright white stripes, and dotted vessels on a pinkish background. (D) Dermoscopic examination of seborrheic keratosis of the areola revealed clear boundary, comedo-like openings, and hairpin vessels. [file Figure_2.TIF]
